# Developmental Nicotine Exposure Alters Synaptic Input to Hypoglossal Motoneurons and Is Associated with Altered Function of Upper Airway Muscles

**DOI:** 10.1523/ENEURO.0299-19.2019

**Published:** 2019-11-14

**Authors:** Lila Buls Wollman, Jordan Clarke, Claire M. DeLucia, Richard B. Levine, Ralph F. Fregosi

**Affiliations:** 1 Department of Physiology, The University of Arizona, Tucson, AZ 85724; 2 Department of Neuroscience, The University of Arizona, Tucson, AZ 85724

**Keywords:** motor neuron, nicotine, perinatal, plasticity, synaptic transmission, whole cell recording

## Abstract

Nicotine exposure during the fetal and neonatal periods [developmental nicotine exposure (DNE)] is associated with ineffective upper airway protective reflexes in infants. This could be explained by desensitized chemoreceptors and/or mechanoreceptors, diminished neuromuscular transmission or altered synaptic transmission among central neurons, as each of these systems depend in part on cholinergic signaling through nicotinic AChRs (nAChRs). Here, we showed that DNE blunts the response of the genioglossus (GG) muscle to nasal airway occlusion in lightly anesthetized rat pups. The GG muscle helps keep the upper airway open and is innervated by hypoglossal motoneurons (XIIMNs). Experiments using the phrenic nerve-diaphragm preparation showed that DNE does not alter transmission across the neuromuscular junction. Accordingly, we used whole cell recordings from XIIMNs in brainstem slices to examine the influence of DNE on glutamatergic synaptic transmission under baseline conditions and in response to an acute nicotine challenge. DNE did not alter excitatory transmission under baseline conditions. Analysis of cumulative probability distributions revealed that acute nicotine challenge of P1–P2 preparations resulted in an increase in the frequency of nicotine-induced glutamatergic inputs to XIIMNs in both control and DNE. By contrast, P3–P5 DNE pups showed a decrease, rather than an increase in frequency. We suggest that this, together with previous studies showing that DNE is associated with a compensatory increase in inhibitory synaptic input to XIIMNs, leads to an excitatory-inhibitory imbalance. This imbalance may contribute to the blunting of airway protective reflexes observed in nicotine exposed animals and human infants.

## Significance Statement

The number one risk factor for sudden infant death syndrome (SIDS) is maternal smoking. While the use of nicotine delivery devices such as e-cigarettes is increasing among women of childbearing age, reflecting the belief that the use of nicotine alone is safer than tobacco, SIDS deaths are not decreasing, suggesting that nicotine is the link between maternal smoking and SIDS. Here, we show that perinatal nicotine exposure alters a major motor pathway responsible for upper airway patency during sleep. We also introduce an animal model that is well suited to probing the mechanisms underlying the link between maternal nicotine consumption and SIDS, and a phenotype that nicely models key aspects of the events believed to give rise to SIDS.

## Introduction

Hypoglossal motoneurons (XIIMNs) innervate the muscles of the tongue, which are critically important in the maintenance of airway patency during breathing (Lowe, 1980). The breathing-related drive to the tongue muscles relies on the appropriate timing and strength of XIIMN output, which is strongly influenced by the balance of excitatory and inhibitory fast-synaptic inputs that the motoneurons receive (Berger, 2011), and a functionally viable neuromuscular junction. Environmental factors, such as exposure to nicotine in the perinatal period, can alter the course of normal nervous system development. This is attributed to nicotine’s action on nicotinic AChRs (nAChRs), which are well known for their role in modulating synaptic transmission in the brain (Wonnacott et al., 2005) and neuromuscular junction (Wood and Slater, 2001). In terms of tongue muscle function in nicotine-exposed human neonates, there are limited although interesting observations. These include an increased incidence of obstructive apneas (Kahn et al., 1994) and hypoplasia and immaturity of XIIMNs (Ottaviani et al., 2006; Lavezzi et al., 2010). In contrast with these limited data from human neonates, there is a considerable body of work on the influence of developmental nicotine exposure (DNE; prenatal exposure with continued exposure in the first week of life) on the structure and function of XIIMNs. For example, XIIMNs from DNE animals have a significantly more complex dendritic arbor than cells from control animals on postnatal days (P)1–P2, but by P3–P4 the arbor is less complex suggesting altered neuronal development over the first week of life (Powell et al., 2016). As for intrinsic properties, XIIMNs from DNE animals are hyperexcitable (Pilarski et al., 2011) and show increased GABAergic inhibition (Jaiswal et al., 2016; Wollman et al., 2018a). We believe that the increase in GABAergic inhibition to nicotine-exposed XIIMNs is consistent with a homeostatic mechanism aimed at mitigating the increased intrinsic excitability. However, a homeostatic response to the increased cell excitability may also include reductions in excitatory synaptic input to XIIMNs.

The experimental results reported here were designed to gain a further understanding of how DNE impacts tongue muscle function at rest and in response to a respiratory challenge, the nature of excitatory synaptic inputs to XIIMNs, and the integrity of the neuromuscular junction. First, tongue muscle function was evaluated *in vivo* by recording the breathing-related tongue muscle EMG before, during and after a period of airway occlusion in lightly anesthetized neonatal rats. Next, since hypoxia and hypercapnia (as occurs during airway occlusion) is associated with increased acetylcholine release (Metz, 1966; Huckstepp et al., 2016), and DNE is known to alter nAChR function throughout the brain (Wonnacott, 1990; Wonnacott et al., 1990; Gentry and Lukas, 2002), we hypothesized that DNE may change how nAChR activation modulates excitatory fast-synaptic inputs to XIIMNs. Accordingly, we evaluated the amplitude and frequency of AMPA receptor-mediated glutamatergic synaptic inputs to XIIMNs at baseline and in response to acute activation of nAChRs. Finally, using the hemidiaphragm-phrenic nerve preparation as a model, we probed the effects of DNE on the integrity of the neuromuscular junction by measuring neuromuscular transmission failure and susceptibility to fatigue.

## Materials and Methods

### Animals

We used a total of 195 Sprague Dawley rat pups of either sex, ranging in age from P1 through P7, which in terms of comparable brain development in humans corresponds roughly to the 23rd week of gestation through birth (Semple et al., 2013). An equal number of control and nicotine exposed animals were used. All neonates were born via spontaneous vaginal delivery from pregnant adult female rats purchased from Charles River Laboratories. Neonates were housed with their mothers and siblings in the animal care facility at the University of Arizona under a 12/12 h light/dark cycle (lights on 7 A.M.), in a quiet room maintained at 22°C and 20–30% relative humidity, and with water and food available ad libitum. All procedures and protocols described were approved by the University of Arizona Institutional Animal Care and Use Committee, and in accordance with National Institutes of Health guidelines.

### DNE

Pregnant Sprague Dawley dams (Charles River Laboratories) were anesthetized with a subcutaneous injection of ketamine (25 mg/kg), xylazine (8.0 mg/kg), and acepromazine (1 mg/kg) and an Alzet 1007D mini-osmotic pump (Alzet Corp.) was implanted subcutaneously on gestational day 5. A subcutaneous injection of buprenorphine (0.5 mg/kg) was given to control postoperative pain. The 28-d pump exposes the pup via the placenta throughout the remainder of gestation (∼16 d), and via breast milk after birth, and these successive pre and postnatal exposures define DNE. The pump was loaded to deliver an average dose of 6 mg/kg/d of nicotine bitartrate. This dose produces plasma cotinine (a metabolic by-product of nicotine) levels in the pups ranging from 60 to 92 ng/ml (Powell et al., 2016), which is comparable to that seen in the plasma of human infants born to mothers who are considered moderate smokers (Berlin et al., 2010). Control animals were obtained from pregnant dams implanted with an Alzet pump filled with saline (sham control). Consistent with our previous studies, there were no systematic differences in measured variables between sham control and animals obtained from pregnant dams that did not undergo pump implantation (true control). Pregnant dams were euthanized on P7 using institutionally and federally-approved procedures.

### *In vivo* studies

**Figure 1.**
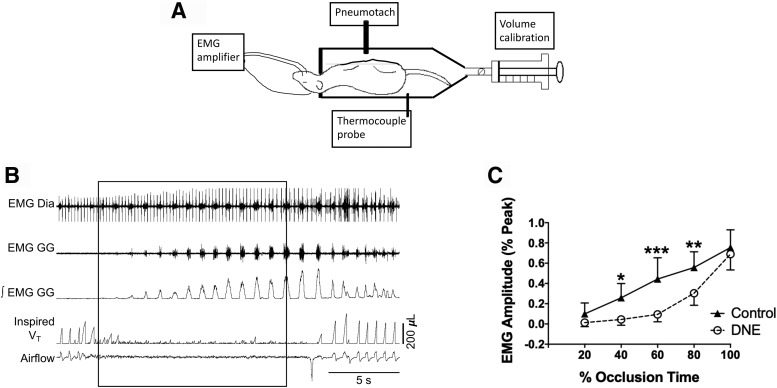
*In vivo* experimental model and EMG response to nasal occlusion. ***A***, Schematic rendition of the *in vivo* experimental preparation, which combines head-out plethysmography and GG EMG recordings in lightly anesthetized neonatal rats, as described in Materials and Methods. ***B***, Example trace showing diaphragm and GG EMG and integrated EMG along with volume and flow traces obtained from the plethysmograph. After 5 min of uninterrupted baseline recordings, a 10- to 15-s nasal occlusion was administered (rectangle). ***C***, GG EMG amplitude during the nasal occlusion. The duration of the nasal occlusion was normalized by dividing the total duration of each occlusion into equal 20% time bins. EMG amplitude was normalized as a percentage of the largest burst recorded (see Materials and Methods). All animals in both groups showed increased GG burst amplitude as the nasal occlusion progressed. *Post hoc* analysis following two-way ANOVA revealed that DNE animals had a significantly blunted amplitude response compared to control at the 40%, 60%, and 80% time bins; *p < 0.05, **p < 0.01, ***p < 0.001.

These studies were designed to test the hypothesis that DNE is associated with decreased drive to the tongue muscles in response to airway occlusion (i.e., the model of an external stressor). Neonatal rat pups ranging in age from P3–P7 were lightly anesthetized with a mixture of ketamine (30 mg/ml), xylazine (6 mg/ml), and acepromazine (3 mg/ml), injected subcutaneously at a volume corresponding to ∼0.35 µl/g body weight. Pain sensitivity was checked via multiple paw pinches initiated 10 min after the time of injection. Supplemental anesthetic was added until paw retraction on pinching was abolished. Whole muscle electromyographic (EMG) activity of the genioglossus muscle (GG, a tongue protrudor muscle) was recorded using fine wire electrodes inserted into the area underneath the tip of the mandible (Fig. 1*A*; Bailey et al., 2005; Rice et al., 2011). An additional hook wire electrode placed into the scruff of the neck near the animal’s ear served as an electrical ground. EMG signals were filtered (30–3000 Hz), amplified (Grass P122 AC amplifiers), and sent to an A/D converter [Cambridge Electronic Design (CED); model 1401], which sampled the signal at a rate of 8333 Hz. After the experiment, animals were killed with an overdose of pentobarbital sodium, and electrode location was confirmed by dissection. Data were accepted only if we could confirm that the wires were in the GG muscle. In four animals, we also inserted pairs of fine wire electrodes into the diaphragm just beneath the lower ribs, to document that overall respiratory motor drive persisted during airway occlusion (Fig. 1*B*).

After implantation of the EMG electrodes, the animal was inserted into a 32 ml head-out plethysmograph in the supine position (Fig. 1*A*). A neck seal was formed with latex, allowing the animal to breathe normally from the room air, with the thorax and abdomen isolated in the sealed chamber. When the animal inhales, its thorax expands, forcing gas out of the sealed chamber. The gas flow entering and leaving the chamber was measured with a pneumotach (Hans-Rudolph) connected to a pressure transducer (Validyne, sensitivity ± 2 cmH2O), and from this we obtained a recording that is proportional to respiratory airflow (Fig. 1*B*, bottom trace). This signal was passed to an analog integrator (Grass) that computed the area under the inspired segment of the curve, providing a measure of the inspired tidal volume (Fig. 1*B*, fourth trace from the top). We calibrated tidal volume by injecting known volumes of gas into the chamber with a 50-µl Hamilton syringe. The pressure, tidal volume and EMG signals were sent to an analog-to-digital converter (CED), displayed in real time on a computer monitor (Spike II software) and stored on a hard drive for subsequent offline analysis. The plethysmograph temperature was maintained between 32°C and 34°C using a temperature probe and heat lamp. This range is within the thermoneutral zone for neonatal rats (Mortola, 1984, 2004; Mortola and Tenney, 1986; Sant'Anna and Mortola, 2003). Although we did not measure body temperature, previous studies show that baseline body temperature is the same in control and DNE rat pups (Ferng and Fregosi, 2015).

**Figure 2.**
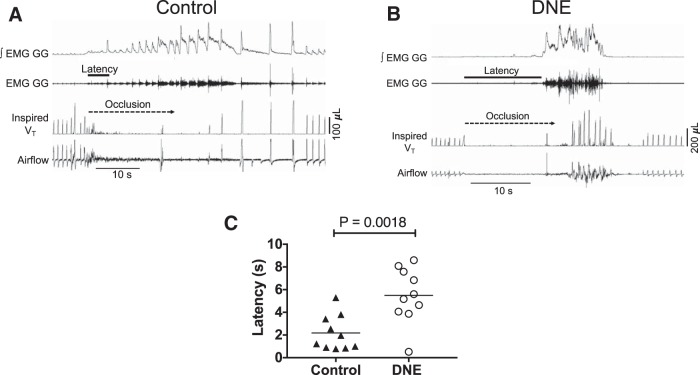
Elapsed time between the onset of nasal occlusion (dashed line) to first discernible GG muscle EMG burst, defined as onset latency. ***A***, ***B*** Recordings from representative control (***A***) and (***B***) DNE pups, as indicated. The length of the solid line under the EMG tracing represents the latency, which is prolonged in the DNE animal following the onset of nasal occlusion. ***C***, Onset latency in nine control and DNE pups. The horizontal lines represent the mean value. An unpaired *t* test revealed a significant difference between the groups (*p* = 0.0018).

After baseline recordings were completed, the animal was challenged with 15 s of nasal occlusion (Fig. 1*B*), resulting in strong breathing efforts but an absence of lung inflation, as well as hypoxia, hypercapnia and acidosis. Measurement of peak EMG activity, tidal volume and breathing frequency throughout the period of nasal occlusion were organized into bins corresponding to five, 20% segments of occlusion time. The EMG activity was normalized in each animal by first detecting the largest burst recorded during the experimental procedure and assigning a value of 1.0 to that burst, which represents the peak activity in each experiment. The average burst amplitude within each 20% time bin was expressed as a fraction of the maximal burst amplitude (Fig. 1*C*). We also measured the time between the onset of nasal occlusion and the onset of the first EMG burst during occlusion, and defined this as the response latency (Fig. 2). Changes in EMG activity during nasal occlusion were analyzed with two-way ANOVA, with time and treatment the main factors. *Post hoc* analysis was by Tukey’s test. The difference in EMG onset latency was tested with the unpaired *t* test; *p* < 0.05 was taken as the threshold for statistical significance (Table 1).

**Table 1.** Statistical tests and significance threshold used for each experiment in each experimental series

Experiment	Statistical test, *post hoc*	Significance threshold
*In vivo* series		
1. Changes to EMG during nasal occlusion	Two-way mixed-model ANOVA with Tukey’s *post hoc* analysis	*p* < 0.05
2. Differences in EMG onset latency	Unpaired Student’s *t* test	*p* < 0.05
3. Autoresuscitation	χ^2^ analysis	*p* < 0.05
*In vitro* series A		
1. Differences in baseline parameters	Two-way mixed model ANOVA with Tukey’s *post hoc* analysis	*p* < 0.05
2. Differences in IEI and amplitude of EPSCs	K–S test of cumulative probability distributions, and three-way mixed model ANOVA with the Holm–Sidak *post hoc* analysis	*p* < 0.05
3. Differences in peak whole cell current	Unpaired Student’s *t* test	*p* < 0.05
*In vitro* series B		
4. Differences in nerve stimulation, muscle stimulation, and estimates of neuromuscular transmission failure	Unpaired Student’s *t* test	*p* < 0.05

**Figure 3.**
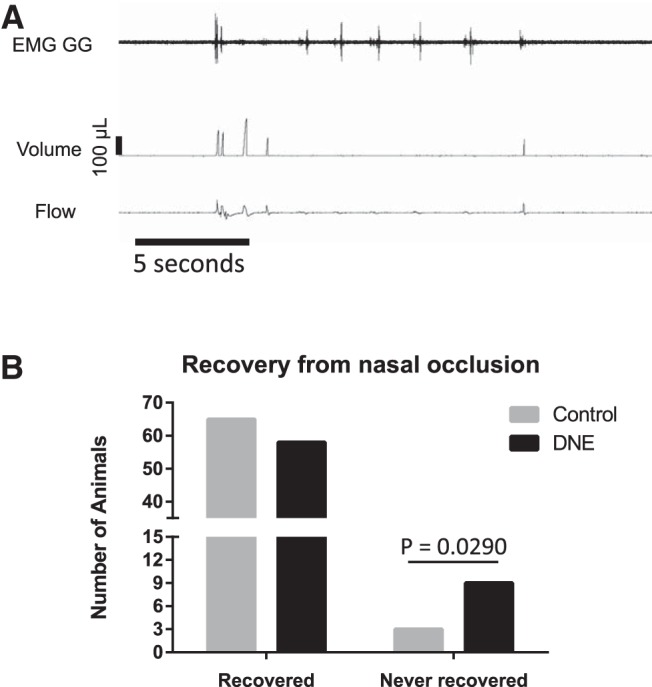
Failure to recover from nasal occlusion. ***A***, An example tracing of one of the 12 animals that exhibited continuous breathing difficulties following nasal occlusion, as explained in Results. ***B***, The number of animals in each treatment group that either recovered from nasal occlusion or failed to recover. Note that of the 12 animals that never recovered, nine were from the DNE group, which is a significant difference by χ^2^ analysis (*p* = 0.0290).

In the course of working out the *in vivo* techniques, we completed 15-s nasal occlusion trials in 135 animals. Only a minority of these animals qualified for the main analysis, which required both high quality plethysmographic and EMG recordings (nine control, 11 DNE); the remainder were not used in the main analysis. However, we did find that 12 of the 135 animals failed to recover from nasal occlusion, and as shown in Figure 3, the majority of these were DNE animals.

### *In vitro* study A, whole-cell voltage clamp recordings of XIIMNs in medullary slice preparations

These experiments were designed to examine the influence of DNE on AMPA-mediated glutamatergic synaptic input to XIIMNs. As indicated below, we studied both spontaneous EPSCs (sEPSCs) and miniature EPSCs (mEPSCs) inputs. This dual focus is important, as sEPSCs reflect both action potential-mediated glutamate release, as well as inputs due to the random, quantal release of glutamate from presynaptic terminals. Moreover, nAChRs are located presynaptically on the soma, dendrites and axon end-terminals of glutamatergic neurons as well as postsynaptically on XIIMNs, and exposure to nicotine could alter nAChRs in all of these locations. Therefore, recording both miniature and spontaneous events can help determine whether the actions of DNE on XIIMNs are presynaptic or postsynaptic.

Pups of either sex were removed from their cages, weighed, anesthetized on ice and decerebrated at the coronal suture. The vertebral column and ribcage were exposed and placed in cold (4–8°C) oxygenated (95% O_2_/5% CO_2_) artificial cerebrospinal fluid (aCSF), composed of the following: 120 mM NaCl, 26 mM NaHCO_3_, 30 mM glucose, 1 mM MgSO_4_, 3 mM KCl, 1.25 mM NaH_2_PO_4_, and 1.2 mM CaCl_2_ with pH adjusted to 7.4 and osmolarity to 300–325 mOsm. The brainstem was extracted and glued to an agar block, rostral surface up, and two to three transverse medullary slices (300–300 microns) containing the hypoglossal motor nucleus were cut in a vibratome (VT1000P, Leica) filled with ice-cold aCSF. The slices were then transferred to an equilibration chamber containing fresh, oxygenated, room temperature aCSF and allowed to recover for 1.5 h before recording.

Equilibrated slices were transferred to a recording chamber maintained at 27°C (TC-324B temperature controller, Warner Instrument Corporation) and perfused with oxygenated (95% O_2_/5% CO_2_) aCSF at a rate of 1.5–2 ml/min. XIIMNs were visualized with an Olympus BX-50WI fixed-stage microscope (40× water-immersion objective, 0.75 N.A.) with differential contrast optics and a video camera (C2741-62, Hamamatsu). Recordings were made with glass pipettes (tip resistance 3–7 MΩ) pulled from thick-walled borosilicate glass capillary tubes (OD: 1.5 mm, ID: 0.75 mm). We used a CsCl based intracellular solution containing the following: 130 mM CsCl, 5 mM NaCl, 2 mM MgCl_2_, 1 mM CaCl, 10 mM HEPES, 2 mM ATP-Mg, 2 mM aucrose, with pH adjusted to 7.2 and osmolarity of 250–275 mOsm. Under these conditions, the chloride reversal potential is ∼0 mV (actual value = –2.8 mV) and therefore both EPSCs and IPSCs are inward at a holding potential of –75 mV. Filled pipettes were attached to a head stage mounted in a micromanipulator (MP-225, Sutter Instrument Company). The head stage was connected to a Multiclamp 700B amplifier, and the signals were digitized with a Digidata 1440A A/D converter (Molecular Devices).

The following procedures pertain to all recordings. First XIIMNs were identified based on their size, shape and location. We targeted the cell soma with the pipette and after a gigaohm seal was achieved the membrane was ruptured by suction. After a 5-min equilibration period to confirm a stable recording, we pharmacologically isolated AMPA receptor-mediated sEPSCs using D-(-)-2-amino-5-phosphonopentanoic acid (AP-5), strychnine hydrochloride, and bicuculline methiodide to antagonize the NMDA receptors the glycine receptors, and the GABA_A_ receptors, respectively (Table 2). In the first set of experiments, sEPSCs were recorded for 3 min at baseline, and then for an additional 3 min during bath application of nicotine (acute nicotine challenge). This protocol was followed by 5 min of washout with aCSF.

**Table 2.** List of drug cocktails used to isolate AMPA receptor-mediated glutamatergic EPSCs and postsynaptic AMPA receptors, with the number of cells used in each condition

Experiments and drugs used	Age	Number of cells (control:DNE)
Glutamate sEPSCs 50 μM AP-5, 10 μM bicuculline, 0.4 μM strychnine + 0.5 μM nicotine	P1–P2 P3–P5	6:6 6:6
Glutamate mEPSCs 50 μM AP-5, 10 μM bicuculline, 0.4 μM strychnine, 1 μM TTX + 0.5 μM nicotine	P1–P2 P3–P5	6:6 6:6
Postsynaptic receptors 50 μM AP-5, 10 μM bicuculline, 0.4 μM strychnine, 1 μM TTX + 2.5 μM AMPA	P1–P2 P3–P5	6:6 6:6

The number of cells studied in each experiment are also shown.

In the second set of experiments we examined the influence of DNE on mEPSCs both before and after an acute nicotine challenge. After a stable recording was achieved, we blocked NMDA, GABA_A_ and glycine receptors, as above, to isolate AMPA-mediated excitatory events. This cocktail was superfused for 3 min, followed by the addition of tetrodotoxin (TTX) for 2 min to block action potential firing (Table 2). AMPA receptor-mediated mEPSCs were recorded at baseline for 3 min, after which nicotine bitartrate was added to the superfusate. mEPSCs were recorded for an additional 3 min in the presence of nicotine, followed by a 5-min washout period.

In a third set of experiments, to evaluate the influence of DNE on postsynaptic AMPA receptors, recordings were again made in the presence of AP-5, strychnine hydrochloride, bicuculline methiodide, and TTX (Table 2). We then bath applied AMPA, to activate postsynaptic AMPA receptors. Under these conditions, activation of the AMPA receptors produces an inward current and the peak of this current was measured. Post synaptic currents were recorded in voltage clamp with Clampex software (Molecular Devices), and analyzed with MiniAnalysis software (Synaptosoft).

### Drugs

Drugs were purchased from Sigma, except for nicotine bitartrate (MP Biomedicals, LLC) and TTX (R&D Chemicals). All drugs were mixed in aCSF on the day of the experiment from previously mixed aliquots that were frozen and stored at 0–2°C. Antagonists were used at concentrations known to be effective based on our previous studies or the literature. Nicotine was used at the highest dose that produced presynaptic effects (increased frequency of sEPSCs in pilot experiments) without producing a significant inward current, which we found to be 0.5 μM. This is important, as pilot studies showed that higher concentrations of nicotine (1, 10, and 100 mM) activates postsynaptic nAChRs and evokes an inward current, that decreases the ability to discriminate sEPSCs/mEPSCs. AMPA was used at a concentration that produced an approximately half maximal response (2.5 μM), based on dose-response experiments previously performed in our lab. The drug solutions were oxygenated and maintained at 27°C and perfused into the recording chamber at a rate of 1.5–2 ml/min.

Data from a total of 72 cells are reported, with 36 cells from DNE neonates and 36 cells from control neonates. Cell numbers for each experiment are summarized in Table 2. The average number of sEPSCs and mEPSCs evaluated per neuron is shown in Table 3. At the end of each experiment, the resting membrane potential and input resistance were measured again to confirm the health of the cell and that the gigaohm seal was still intact. Because of the morphologic differences seen in neurons from DNE animals aged P1–P2 compared to age P3–P5 (Powell et al., 2016), we analyzed our data within these two age groups. For all EPSCs, the interevent interval (IEI) and peak amplitude were measured during the minute before the nicotine challenge, and throughout the second and third minutes of the challenge. For mEPSCs, rise time was measured at these same time points. There were no differences for any of these variables between the second and third minutes of nicotine challenge, so data recorded in the third minute was used for analysis. Lastly, we measured the peak of the whole cell inward current evoked by stimulation of postsynaptic AMPA receptors with bath applied AMPA (For example, see Fig. 9*A*). 

**Table 3.** Number of sEPSCs and mEPSCs recorded per neuron at baseline and during acute nicotine application

	Control	DNE
sEPSC		
P1–P2BaselineAcute nicotine	65 ± 2374 ± 59	91 ± 38187 ± 105
P3–P4BaselineAcute nicotine	145 ± 107211 ± 211	116 ± 8481 ± 60
mESPCs		
P1–P2BaselineAcute nicotine	28 ± 1438 ± 20.8	28 ± 2261 ± 39
P3–P4BaselineAcute nicotine	18 ± 1331 ± 15	41 ± 3833 ± 27

Values are mean ± SD.

### *In vitro* study B, hemidiaphragm-phrenic nerve preparation

To estimate the influence of DNE on the integrity of the neuromuscular junction, and as a substitute for the hypoglossal nerve-tongue neuromuscular junction (see Discussion), we used neonatal rat (P1–P5) phrenic nerve-hemidiaphragm preparations. Diaphragm muscle was excised and secured in a dish perfused with warmed (37°C), oxygenated Krebs solution, containing the following: 123 mM NaCl, 26 mM NaHCO^-^_3_, 30 mM glucose, 1 mM MgSO_4_, 3 mM KCl, 1.25 mM NaH_2_PO_4_, and 1.2 mM CaCl, gassed with 95% O_2_/5% CO_2_, with pH adjusted to 7.45–7.5. Force was measured with a transducer (Kent Scientific) attached to the central tendon of the hemidiaphragm, and the phrenic nerve was drawn into a suction electrode, which was referenced to a bath ground and connected to a stimulator (Grass S88). Two silver/silver-chloride discs were pinned to the bath on either side of the muscle strip and connected to a second channel on the stimulator for direct depolarization of the muscle fibers, bypassing the neuromuscular junction. Force and the output of the stimulator were digitized and monitored on a computer using Spike II hardware and software (CED).

To measure muscle twitch force and contraction speed in response to direct muscle stimulation we delivered single, 0.2-ms supramaximal pulses to the bath electrodes. We also measured the decline in force following 5 min of intermittent, supramaximal direct muscle stimulation, while in another set of animals we stimulated the phrenic nerve. Both muscle and nerve were stimulated with 0.2-ms pulses, delivered in 330-ms trains at 40 Hz, with a train delivered every 2 s. In additional sets of animals, we estimated the contribution of neuromuscular transmission to force loss by applying stimulus trains to the phrenic nerve, as above, while superimposing direct muscle stimulation every 15 s. Neuromuscular transmission failure was computed by comparing the force loss during nerve stimulation with that evoked by direct muscle stimulation (Fig. 10*C*), as described by others (Aldrich et al., 1986; Fournier et al., 1991). Force decline due to neuromuscular transmission failure = (force loss during nerve stimulation – force loss during muscle stimulation)/(1 – force loss during muscle stimulation).

### Statistics

A brief summary of the statistical analysis used in each of the three experimental series is given in Table 1. For *in vivo* experiments, changes in EMG activity during nasal occlusion were analyzed with two-way ANOVA, with time and treatment the main factors. *Post hoc* analysis was by Tukey’s test. The difference in EMG onset latency was tested with the unpaired *t* test; *p* < 0.05 was taken as the threshold for statistical significance (Table 1). Differences in the number of failed autoresuscitations was compared between groups using a χ^2^ analysis (Table 1).

### *In vitro* study A, whole-cell voltage clamp recordings of XIIMNs in medullary slice preparations

Differences in baseline variables including age, weight, resting membrane potential, and input resistance were evaluated between treatment groups, and between age groups within a treatment group, by comparing the means from each group with a two-way ANOVA, followed by Tukey’s *post hoc* test for multiple comparisons (Table 1).

We analyzed the IEI and amplitude for both sEPSCs and mEPSCs in two ways (Table 1). First, the IEI and amplitude of all sEPSCs/mEPSCs from all cells within an age or treatment group were used to construct a cumulative probability distribution using Prism (GraphPad Software, Inc.; For example, see Figs. 6, 8). We expressed the cumulative probability as fractions ranging from 0 to 1, such that a value of 0.5 defines the midpoint of the normalized EPSC amplitude or frequency distribution. In these graphs, the *y*-axis value is the fraction of events that lie at or below the corresponding *x*-axis value. To compare the IEI and amplitude distributions between baseline conditions and during acute nicotine application, we performed a two-sample Kolmogorov–Smirnov (K–S) test using SPSS (IBM).

Second, in each cell we recorded the median value of IEI and the event amplitude for every event, both at baseline and during acute nicotine challenge in both age and treatment groups. We then ran a separate mixed model, three-way factorial ANOVA for each of these variables, with the main factors the treatment group (control vs DNE), age (P1–P2 vs P3–P5) and experimental condition (baseline vs acute nicotine challenge). When the ANOVA was significant, we conducted *post hoc* analyses using the Holm–Sidak multiple comparisons test, with the alpha level set at *p* < 0.05. We used an unpaired *t* test to compare the mean peak current in control and nicotine exposed cells (Table 1). We note that this approach is considerably more conservative than the analysis of cumulative probability distributions, which is the most popular technique for analyzing excitatory and inhibitory synaptic potentials (Henze et al., 1997). This is because probability distributions typically pool all events from all cells into a single distribution, which inflates the statistical power.

### *In vitro* study B, hemidiaphragm-phrenic nerve preparation

Average values for force loss in response to nerve stimulation, muscle stimulation, and estimates of neuromuscular transmission failure in control and DNE animals were compared with the unpaired *t* test. As above, *p* < 0.05 was the threshold for statistical significance (Table 1). Average data are reported as the mean ± SD throughout the manuscript.

## Results

### *In vivo* studies: influence of DNE on tongue muscle EMG activity in response to airway obstruction

We began our study by assessing the impact of DNE on neural drive to the GG muscle of the tongue, which is innervated by XIIMNs and plays a major role in keeping the upper airway open (Remmers et al., 1978). We did this by challenging the system with a 15 s nasal occlusion, initiated during the expiratory period. Airway occlusion in neonates typically results in a strong increase in drive to the muscles of breathing. The representative recordings in Figure 1*B* show that nasal occlusion is associated with a monotonic increase in the EMG activity of both the diaphragm and the GG. The group average data (Fig. 1*C*) show that the increase in GG EMG activity during occlusion was significantly blunted in the DNE animals, consistent with a reduced excitation of XIIMNs. In addition to the reduced amplitude of the GG EMG during occlusion, the onset latency was longer in DNE animals (*p* < 0.001), as shown in the representative recordings in Figures 2*A*,*B*, with individual and mean values shown in Figure 2*C*. This observation is consistent with a DNE-mediated blunting of excitatory drive to hypoglossal motoneurons during airway occlusion.

As indicated in Materials and Methods, we did the nasal occlusion test in 135 neonates and noted that 12 of the 135 never recovered, and instead entered a state characterized by terminal gasping. The pattern of breathing in animals that did not recover most often approximated that shown in Figure 3*A*, in that long periods of apnea were terminated following bursts of GG activity. Figure 3*A* also shows that, at times, multiple bursts of GG EMG activity were required to terminate an apnea. These observations are consistent with upper airway obstruction, as the bursting pattern of GG EMG activity suggests that the XIIMNs were receiving drive from the respiratory central pattern generator but airflow was not detected. Interestingly, of the 12 neonates that entered this state of unstable breathing, nine were DNE animals (Fig. 3*B*). This difference in the ability to survive nasal airway occlusion was statistically significant by χ^2^ analysis (χ statistic = 3.594, *z* score = 1.896, *p* = 0.0290), with an adjusted odds ratio of 3.5.

### *In vitro* study A, whole-cell voltage clamp recordings of XIIMNs in medullary slice preparation

#### Influence of DNE on age, weight, Vm, and input resistance

The age and weight of the animals used in these experiments are shown in Table 4. The age of the animals studied in each treatment group was the same, eliminating age bias in the results. The older animals were heavier, as expected, but there were no differences in body weight between control and DNE animals in either age group. There were also no effects of age or treatment for either Vm or input resistance (Table 4). We note that the values for input resistance are higher than values recorded with standard intracellular solutions, but are consistent with values recorded with Cs-based intracellular solutions, as used here.

**Table 4.** Age, weight, resting membrane potential (mV), and input resistance (Rin) of XIIMNs from control and DNE animals

	Control	DNE	*p* value	*n* (control:DNE)
Age (d)				
P1–P2 P3–P5	1.7 ± 0.23.4 ± 0.1	1.4 ± 0.13.7 ± 0.2	*p* = n/s*p* = n/s	18:1818:18
Weight (g)				
P1–P2 P3–P5	7.72 ± 0.3110.53 ± 0.44***	7.4 ± 0.110.8 ± 0.3***	*p* = n/s*p* = n/s	18:1818:18
Vm				
P1–P2 P3–P5	–49 ± 3–48 ± 2	–48 ± 2–47 ± 2	*p* = n/s*p* = n/s	18:1818:18
Rin (MΩ)				
P1–P2 P3–P5	221 ± 49185 ± 41	184 ± 23189 ± 42	*p* = n/s*p* = n/s	4:44:4

**Table 5.** Mean values for amplitude of glutamatergic sEPSCs, mEPSCs, and mEPSC rise time at baseline and during acute nicotine challenge

	Baseline	Acute nicotine challenge
sEPSC amplitude (pA)Control:		
P1–P2P3–P5DNE:P1–P2P3–P5	–15.2 ± 2.7–15.5 ± 2.3 –18.5 ± 7.4–16.4 ± 2.8	–16.0 ± 3.2–15.7 ± 3.9 –18.3 ± 8.9–14.8 ± 2.9
mEPSC amplitude (pA)Control:		
P1–P2P3–P5DNE:P1–P2P3–P5	–16.2 ± 2.6–14.9 ± 3.0 –16.0 ± 2.9–17.5 ± 1.9	–14.6 ± 2.6–16.3 ± 3.7 –15.6 ± 3.8–17.4 ± 1.9
mEPSC rise time (ms)Control:		
P1–P2P3–P5DNE:P1–P2P3–P5	2.1 ± 0.2 2.0 ± 0.1 2.1 ± 0.2 1.8 ± 0.1	2.1 ± 0.2 2.2 ± 0.1 2.0 ± 0.1 2.0 ± 0.1

There were no significant differences in any of these variables.

#### Influence of DNE on the frequency and amplitude of glutamatergic sEPSCs in brainstem slices

**Figure 4.**
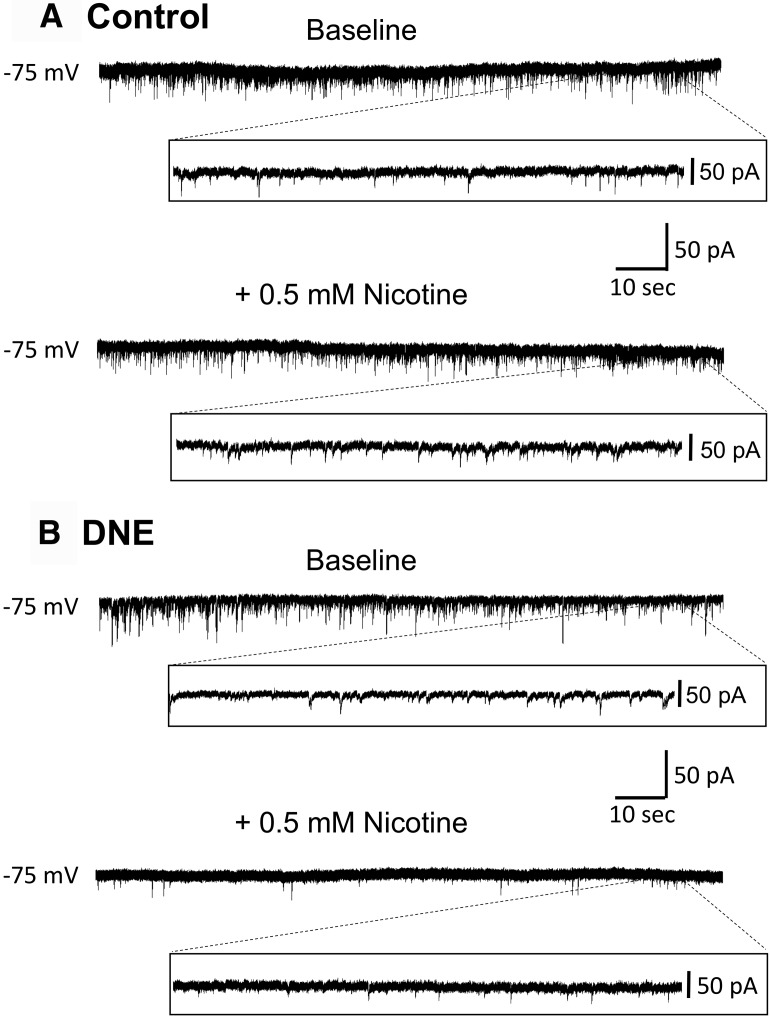
Example traces of AMPA receptor-mediated sEPSCs recorded from XIIMNs. ***A***, ***B***, Representative traces of pharmacologically isolated sEPSCs from a control animal (***A***) and a DNE animal (***B***), both studied on P4. Each trace shows the entire 3-min recording period at baseline (upper trace in each panel) and during acute nicotine application (bottom trace in each panel). Inset panels are an expanded view, showing 10 s of the recording at the end of each trace. Note that the size and frequency of events at baseline is similar in the control and DNE animals. In contrast, with acute nicotine challenge (bottom trace in each panel) the DNE cell shows a decrease in sEPSC frequency, whereas there is a modest increase in the control cell.

**Figure 5.**
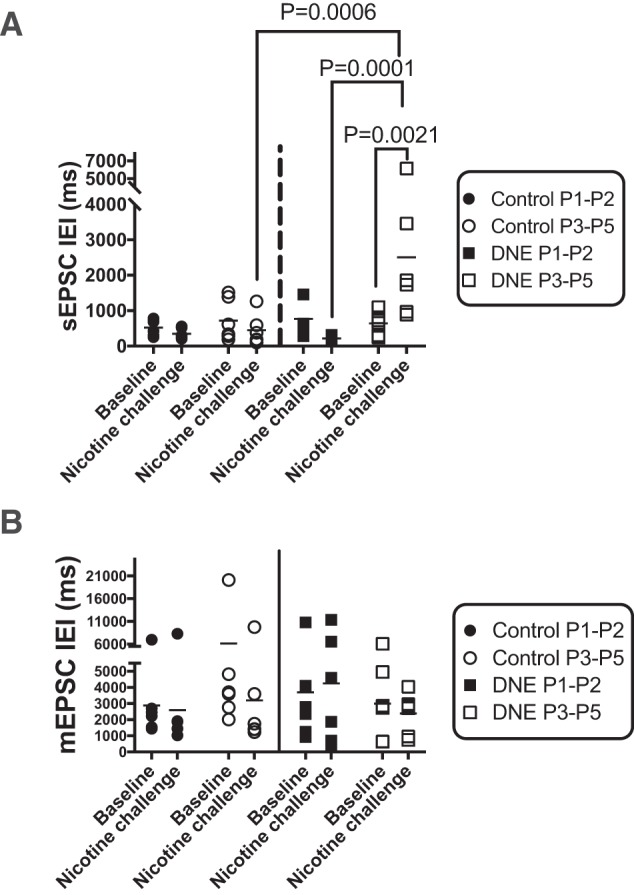
Individual and average IEI of sEPSCs and mEPSCs at baseline and during acute nicotine challenge. ***A***, Individual and average IEI of AMPA receptor-mediated sEPSCs under baseline conditions and following acute nicotine challenge. Note that in control cells (circles, left of the vertical line) there was a slight although non-significant decline in IEI (i.e., an increase in frequency) on both P1–P2 (filled circles) and P3–P5 (open circles). The IEI in response to an acute nicotine challenge in cells from DNE animals was age-dependent (squares, right of the vertical line). Note that on P1–P2, nicotine challenge decreased the IEI, although as in controls this trend was not significant. Surprisingly, on P3–P5 acute nicotine challenge significantly increased the IEI (*p* = 0.0021). Moreover, the mean value for IEI in P3–P5 DNE cells during acute nicotine challenge is significantly different from corresponding data in P1–P2 DNE cells (*p* = 0.0001), and in P3–P5 cells from control animals. ***B***, Individual and average IEI of AMPA receptor-mediated mEPSCs under baseline conditions and following acute nicotine challenge. Note that in the P1–P2 group, both control and DNE cells show a trend toward a decrease in IEI with acute nicotine challenge; however, this was not significant. Analysis of average IEI from control and DNE neurons at P3–P5 shows no differences at baseline and no change in frequency with acute nicotine challenge.

To evaluate the influence of DNE on network level AMPA receptor-mediated synaptic events under baseline conditions, we evaluated differences in the IEIs and the amplitude of sEPSCs in control and DNE cells. The upper traces in Figures 4*A*,*B* are example recordings of glutamatergic sEPSCs from one control and one DNE cell under baseline conditions; both recordings were obtained from a P4 animal. There are no obvious differences in the traces from control and DNE cells at baseline, which is consistent with comparisons of the median data showing no significant differences for baseline sEPSC IEI (Fig. 5*A*) or amplitude (Table 5) between control and DNE cells, in either of the age groups.

**Figure 6.**
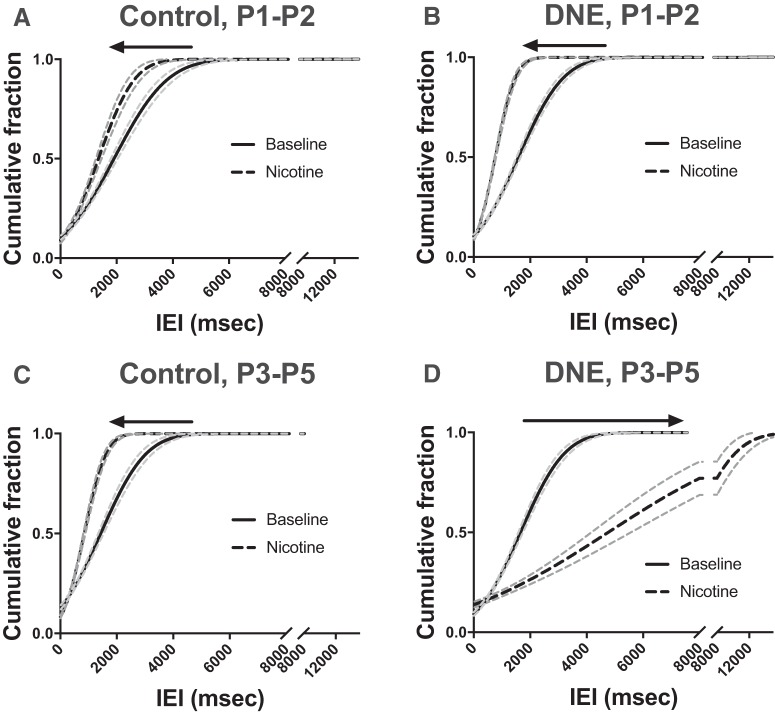
DNE alters the modulation of glutamatergic sEPSCs in response to an acute nicotine challenge but only in cells from pups aged P3–P5. Cumulative probability distributions of glutamatergic sEPSC IEIs in control and DNE cells, at baseline and during acute nicotine challenge. At P1–P2 (***A***, ***B***), acute nicotine challenge with 0.5 μM nicotine (black dashed lines) caused a left shift, toward shorter IEIs, of glutamatergic sEPSCs in both control and DNE cells [control, *p* = 0.049 (***A***) DNE, *p* < 0.0001 (***B***)]. In cells from P3–P5 animals (***C***, ***D***), acute nicotine challenge caused a left shift, toward shorter IEIs in control cells (*p* < 0.0001; ***C***), but the distribution shifted to the right, toward longer IEIs in the DNE cells (*p* = 0.012; ***D***). Arrows indicate the direction of the shift with acute nicotine challenge and indicates significant differences with K–S test (see Materials and Methods). Dotted gray lines indicate the 95% confidence intervals of each curve.

We then studied the influence of an acute nicotine challenge on the frequency and amplitude of sEPSCs. The tracings in Figure 4 (recordings labeled +0.5 μM nicotine) show a modest increase in sEPSC frequency with nicotine challenge in the control cell, while the DNE cell showed little change in amplitude, but a decrease in frequency. Examination of cumulative probability curves in animals aged P1–P2 (Fig. 6*A*,*B*) show that the distributions shifted to the left, to lower IEIs (higher frequency) with acute nicotine challenge in both control (*p* = 0.049, based on 675 and 749 events at baseline and with acute nicotine challenge, respectively) and DNE cells (*p* < 0.0001, 871 events at baseline, 1170 events with nicotine challenge). In animals aged P3–P5, nicotine challenge again shifted the cumulative probability distributions to the left in control animals (*p* < 0.0001, based on 570 and 1271 events analyzed at baseline and during nicotine challenge, respectively; Fig. 6*C*). However, in the P3–P5 DNE animals acute nicotine challenge shifted the curve to the right, indicating a slowing, rather than an increase in sEPSC frequency (*p* = 0.012, based on 678 and 501 events analyzed at baseline and during nicotine challenge, respectively; Fig. 6*D*).

We also analyzed the group data for all events by first computing the median value of all events in each cell, followed by calculation of the grand median value within each of the four age and treatment groups (Fig. 5*A*). The group median data show that in control cells, acute nicotine challenge had no significant effect on the IEI at either age (Fig. 5*A*). In contrast, whereas acute nicotine challenge had no significant effect on DNE cells at ages P1–P2, it was associated with an increase in sEPSC IEI in cells from animals aged P3–P5, indicating a significant decrease in the frequency of sEPSCs (*p* = 0.0021; Fig. 5*A*). In addition, the change in IEI in the DNE cells from P1–P2 and P3–P5 animals is significantly different (*p* = 0.0001; Fig. 5*A*). The IEI in DNE cells from P3–P5 pups was also different from the IEI in cells from P3–P5 control cells (*p* = 0.0006; Fig. 5*A*). As with control cells, nicotine challenge had no effect on sEPSC amplitude in cells from DNE animals at either age, and this was true whether we analyzed the entire population of events with cumulative probability analyses (data not shown), or with ANOVA based on the average data in each age and treatment group (Table 5).

#### Frequency, amplitude, and rise time of glutamatergic mEPSCs

**Figure 7.**
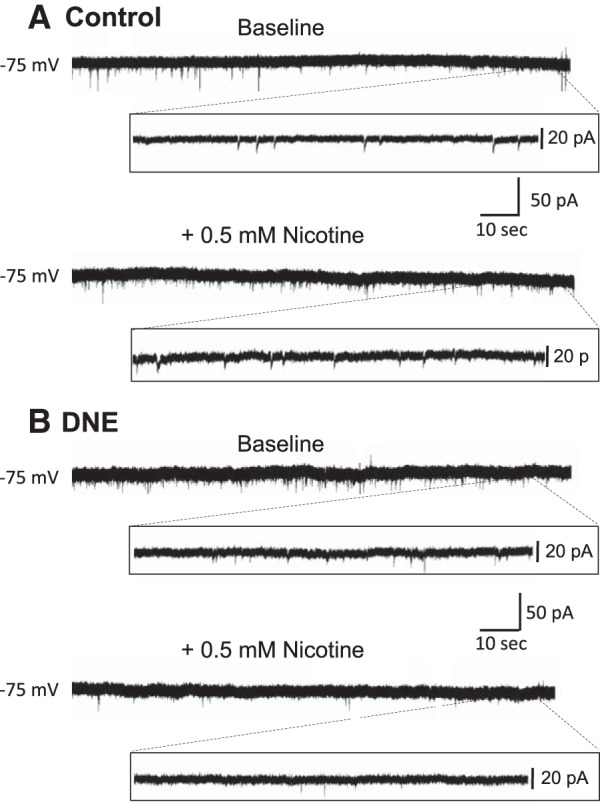
Influence of DNE on AMPA receptor-mediated mEPSCs recorded from XIIMNs. ***A***, ***B***, Representative traces of pharmacologically isolated mEPSCs from a control animal (***A***) and a DNE animal (***B***) studied on P4. Each trace shows the entire 3 min of recording at baseline (top trace) and during acute nicotine application (bottom trace). Inset panels show an expanded view of 10 s at the end of each trace, as in Figure 4. Note that the size and frequency of events is similar in the control and DNE cell at baseline. However, during acute nicotine challenge, mEPSC frequency increased in control cells but not in DNE cells.

We also evaluated how DNE affects the frequency, amplitude and rise time of AMPA receptor-mediated mEPSCs, which represent the random, quantal release of glutamate from presynaptic terminals. The upper traces in Figures 7*A*,*B* are mEPSCs recorded under baseline conditions, while the lower traces were recorded during acute nicotine challenges. Figures 7*A*,*B* are from a control and DNE animal, both studied on P2.

**Figure 8.**
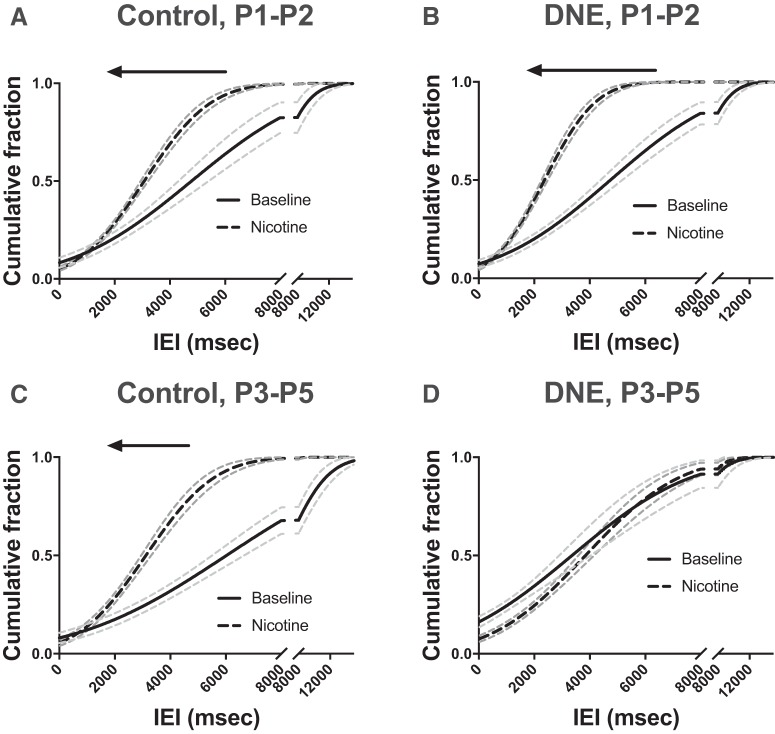
Cumulative probability distributions of glutamatergic mEPSC IEIs in control and DNE cells, at baseline and during acute nicotine challenge. In cells from animals aged P1–P2 (***A***, ***B***), acute nicotine challenge with 0.5 μM nicotine (black dashed lines) caused a left shift, toward shorter IEIs, of glutamatergic mEPSCs in both control (*p* < 0.0001; ***A***) and DNE cells (*p* < 0.0001; ***B***). In cells from animals aged P3–P5 (***C***, ***D***), acute nicotine challenge caused a significant left shift of glutamatergic mEPSCs in control cells (*p* = 0.004; ***C***), but there was no change in the distribution of IEIs in DNE cells (*p* = 0.066; ***D***). Arrows indicate the direction of the shift with acute nicotine challenge and indicates significant differences with K–S test (see Materials and Methods). Dotted gray lines indicate the 95% confidence intervals of each curve.

Examination of cumulative probability curves in animals aged P1–P2 (Fig. 8*A*,*B*) show that the distributions shifted to the left, to lower IEIs (higher frequency) with acute nicotine challenge in both control (*p* < 0.0001, based on 168 and 221 events at baseline and with acute nicotine challenge, respectively) and DNE cells (*p* < 0.0001, 169 events at baseline, 361 events with nicotine challenge). In animals aged P3–P5, nicotine challenge again shifted the cumulative probability distributions to the left in control animals (*p* = 0.004, based on 91 and 164 events analyzed at baseline and during nicotine challenge, respectively; Fig. 8*C*). However, in the P3–P5 DNE animals acute nicotine challenge did not affect the cumulative probability distribution of mEPSCs significantly (*p* = 0.066, based on 186 and 202 events analyzed at baseline and during nicotine challenge, respectively; Fig. 8*D*).

Analysis of the group median data did not reveal significant differences in either the frequency (Fig. 5*B*) or amplitude (Table 5) of mEPSCs in either control or DNE cells, both at baseline and with acute nicotine challenge. Similarly, there were no significant differences for mEPSC rise time either between treatment groups at baseline, or between baseline and nicotine challenge within a treatment group (Table 5).

#### Influence of DNE on postsynaptic AMPA receptors

**Figure 9.**
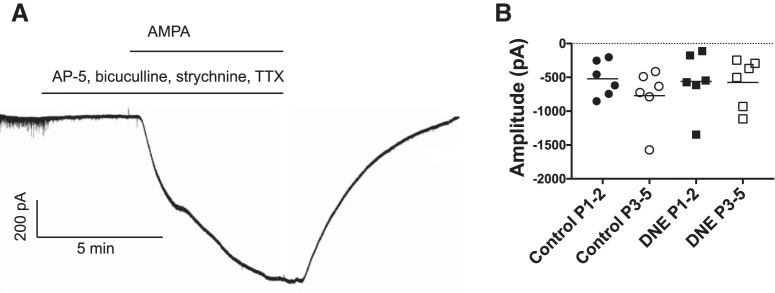
Activation of postsynaptic AMPA receptors in control cells and DNE cells. ***A***, Representative trace of the AMPA receptor-mediated inward current in XIIMNs from a control animal at P1**. *B***, Individual values for the peak inward current in response to bath application of AMPA. Mean values within each treatment group are indicated by the horizontal lines. There were no differences in the magnitude of the postsynaptic inward current either within or between treatment groups.

To gain a more complete understanding of DNE-mediated effects on AMPA receptor function, we obtained recordings from XIIMNs and measured the peak inward current that was produced by bath application of 2.5 μM AMPA in the presence of AP-5, strychnine hydrochloride, bicuculline methiodide, and TTX (Table 2). Figure 9*A* shows an example trace of the inward current that results from activation of the postsynaptic AMPA receptors with bath application of 2.5 μM AMPA in a control cell from a P1 animal. The average peak amplitude of the inward current was the same in DNE and control cells in both age groups, and there were no differences in current amplitude with age in either control cells or DNE cells (Fig. 9*B*).

### *In vitro* study B, influence of DNE on diaphragm muscle contractile properties and the susceptibility to fatigue

**Figure 10.**
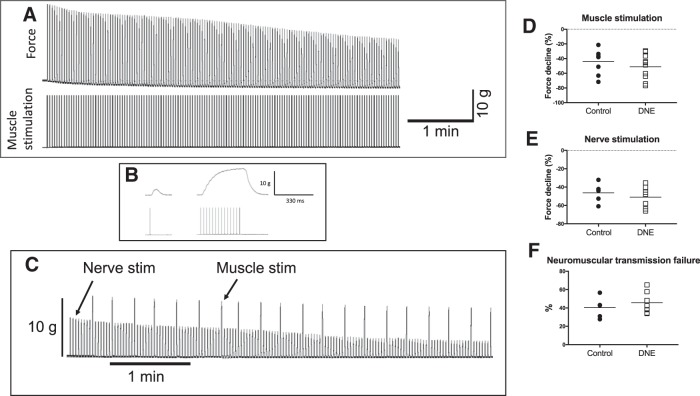
Influence of DNE on diaphragm muscle fatigue and neuromuscular transmission failure (NTF) during repetitive muscle or phrenic nerve stimulation in diaphragm phrenic nerve-muscle strips. ***A***, Representative force recording over a 5-min period of direct muscle stimulation. Fatigue was quantified as the % of the maximum force remaining at the end of the 5-min stimulation period (see Materials and Methods). ***B***, Muscle twitch produced by a single stimulus pulse, and an expanded view of the muscle force produced by a 330-ms train of stimulation pulses, as described in Materials and Methods. An identical protocol was used when phrenic nerve stimulation was used to assess the magnitude of force decline. ***C***, Representative force recording during 5 min of repeated phrenic nerve stimulation with superimposed direct muscle stimulation every 15 s. NTF was calculated as: the percentage force declines in each control and DNE preparation subjected to either muscle (***D***) or phrenic nerve (***E***) stimulation. ***F***, Percentage neuromuscular transmission failure in control and DNE preparations. Horizontal lines in ***D–F*** indicate the mean. There were no significant differences in the percentage force loss between control and DNE preparations with either muscle or nerve stimulation, or the force loss due to neuromuscular transmission failure.

Contractile properties (mean ± SD) including peak twitch tension (control, 2.5 ± 1.2 g; DNE 2.6 ± 1.3 g), time to peak tension (Control, 50 ± 10 ms; DNE 60 ± 10 ms), and ½ relaxation time (control, 70 ± 20 ms; DNE 60 ± 20 ms) were the same in diaphragm strips from control and DNE animals. We note that our values for each of these variables are similar to those reported in 7-d-old neonatal rats (Zhan et al., 1998). Figure 10*A* is an example of the force decline recorded in diaphragm muscle strips following repetitive trains of phrenic nerve stimulation. Figure 10*B* shows an expanded view of the force evoked by a single twitch, and by a 330 ms train of impulses. Figure 10*C* is an example of the protocol used to estimate the contribution of neuromuscular transmission failure to the decline in force. Note that direct muscle stimulation was superimposed on phrenic nerve stimulation approximately every 15 s, and the difference in force between nerve and muscle stimulation was used to compute an index of neuromuscular transmission failure (see Materials and Methods). The force loss following repetitive stimulation of muscle directly (Fig. 10*D*) or the phrenic nerve (Fig. 10*E*) was the same in control and DNE preparations. Similarly, the percentage of the force decline attributable to neuromuscular transmission failure was the same in both treatment groups (Fig. 10*F*). Thus, DNE does not impair the physiology of the neuromuscular junction in diaphragm muscle.

## Discussion

Inadvertent obstruction of the airway (e.g., an infant’s head covered by bedding) is accompanied by an increase in blood CO_2_ and a decrease in O_2_, leading to an increase in the release of several neurotransmitters, including ACh (Metz, 1966). The increased release of excitatory neurotransmitters helps support the respiratory motor response to chemoreceptor stimulation. Here, we found that nicotine exposed neonatal rats had a delayed and blunted GG muscle motor response to nasal airway occlusion, consistent with an attenuation of excitatory drive to XIIMNs under these conditions. We then used an *in vitro* approach to examine excitatory synaptic input to XIIMNs both under baseline conditions and in response to an acute nicotine challenge, which mimics the stimulation of nAChRs that accompanies the release of endogenous ACh, or an increase in central nicotine levels secondary to tobacco smoking or the use of nicotine delivery devices. The key finding is that in P3- to P5-d-old animals, an acute nicotine challenge evoked a marked increase in excitatory synaptic input to XIIMNs in control animals, while the frequency decreased in the DNE pups. Together with previous work showing that nicotine challenge increased GABAergic inhibitory input to XIIMNs (Neff et al., 2003; Jaiswal et al., 2016; Wollman et al., 2018a), these observations suggest that DNE shifts the excitatory-inhibitory balance in XIIMNs toward inhibition in response to a nicotine challenge. This is a deviation from the normal response of neural systems, wherein neuronal spiking activity is maintained within homeostatic limits by careful adjustments in the balance of excitatory and inhibitory synaptic inputs to a neuron or network of neurons (Turrigiano, 2012; Wenner, 2014). Below, we discuss how alterations in nAChR function and/or altered development of XIIMNs may underlie the observation that DNE blunts the GG motor response to nasal occlusion and reduces the likelihood of surviving an occlusion.

### DNE blunts the GG motor response to airway obstruction

Previous work has mainly used *in vitro* techniques to evaluate how DNE alters neurotransmission and the development of hypoglossal motoneurons, which innervate the tongue muscles. In contrast, we are unaware of any studies on the influence of DNE on the control of tongue muscle activity *in vivo*. Here, we show that DNE blunts the magnitude and delays the activation of the GG EMG during imposed nasal occlusions. Although we are unable to establish the mechanisms underlying these observations, there are clues from previous work. First, nasal occlusion prevents lung inflation, and the absence of inflation releases hypoglossal motoneurons from inhibition, leading to increased GG activity, at least in adult rodents (Bailey et al., 2001). Second, the previously described reduction in chemosensitivity with DNE in neonatal rodents (St-John and Leiter, 1999; Fewell et al., 2001; Simakajornboon et al., 2004; Hafström et al., 2005; Eugenín et al., 2008; Mahlière et al., 2008; Huang et al., 2010) may explain the reduced GG activity during nasal occlusion, as combined hypoxia and hypercapnia become progressively more severe as the occlusion continues and DNE animals are not as well equipped to initiate chemoreceptor-mediated activation of hypoglossal motoneurons. The mechanism of the blunted ventilatory response to chemoreceptor stimulation in nicotine-exposed animals is unknown but could be due to effects on peripheral and central chemoreceptor signaling, and/or a reduced respiratory motor response to the chemoreceptor afferent input. Third, *in vitro* studies show that DNE is associated with various disruptions in the growth, intrinsic membrane properties and synaptic inputs to hypoglossal motoneurons (Robinson et al., 2002; Pilarski et al., 2011; Jaiswal et al., 2013; Powell et al., 2016; Vivekanandarajah et al., 2016). It is likely that each of these factors contribute in some way to the blunted GG response to nasal occlusion in nicotine exposed pups.

We also showed that the majority of pups that failed to recover from nasal occlusion were nicotine exposed. Animals that were used to obtain the main experimental data sets had to meet certain criteria in order for their responses to be analyzed. Criteria included reestablishing inspiratory flow, tidal volume, breathing frequency and EMG activity to baseline levels following nasal occlusions. A total of 125 animals met these criteria, while 12 did not. Of great interest is that nine out of the 12 animals that failed to recover were nicotine exposed. These findings suggest that DNE may increase the probability that a neonate experiencing an airway occlusion will fail to reestablish a normal breathing pattern, leading to hypoxia and acidosis. An important caveat that envelopes ventilatory measures in neonatal animals is that the stress of maternal separation may interact with DNE and contribute to the altered GG EMG responses observed. Indeed, impaired volitional motor control in DNE animals is exacerbated by maternal separation (Bassey and Gondre-Lewis, 2019).

### Influence of DNE on AMPA receptor-mediated glutamatergic neurotransmission

Surprisingly, recordings done under baseline conditions did not reveal differences in the frequency or amplitude of sEPSCs or mEPSCs between control cells and DNE cells at either age. We also assessed postsynaptic receptor function with bath application of AMPA in synaptically isolated neurons. There were no differences in peak AMPA current in control or DNE cells in either age group, and there were no age-dependent changes within a treatment group for any of these variables. We note that this approach stimulates both synaptic and extra-synaptic AMPA receptors which may respond differently to DNE. Nevertheless, when taken together with the data showing no change in mESPC amplitude with DNE, the findings indicate that under baseline conditions, DNE does not alter glutamatergic synaptic input to XIIMNs, or the postsynaptic response to AMPA.

An important caveat underlying these observations is that previous work showed that the frequency of AMPA receptor-mediated glutamatergic sEPSCs was lower in XIIMNs from DNE cells compared to control cells (Pilarski et al., 2011). However, the latter experiments were done for a different purpose, with recordings made in the setting of elevated extracellular potassium (9 mM) in thick brainstem slices. Moreover, because the cells were bursting under these conditions, the excitatory inputs could only be discerned in the interburst interval, they were not isolated pharmacologically (i.e., inhibitory transmission was intact) and the response to acute nicotine challenge was not examined. Accordingly, the present results extend these earlier findings.

In many physiologic systems underlying pathology is not obvious under baseline conditions but can be revealed when the system is stimulated. It is well known that stimulation of nAChRs on glutamatergic neurons leads to increased release of glutamate (Wonnacott et al., 1990; Wonnacott, 1997; Gentry and Lukas, 2002). Importantly, chronic nicotine exposure is associated with both an upregulation and long-term desnensitization of nAChRs in many neuron types (Wonnacott, 1990; Gentry and Lukas, 2002), including XIIMNs (Pilarski et al., 2012; Wollman et al., 2016). Accordingly, we also studied glutamatergic synaptic transmission while nAChRs were activated with an acute nicotine challenge. Whereas nicotinic stimulation of glutamatergic synaptic transmission was the same in control and DNE pups studied on P1 and P2, there were significant treatment effects in P3–P5 animals. In P3–P5 control animals, acute nicotine challenge increased the frequency of both sEPSCs and mEPSCs, while the frequency of sEPSCs decreased in the DNE animals. Importantly, the stimulation of mEPSCs by acute nicotine challenge in control preparations indicates that nicotine is likely acting on the presynaptic terminals of glutamatergic neurons that impinge on the motoneurons. The stimulation of sEPSC frequency could involve other sites of nicotine’s action, including the soma or dendrites of glutamatergic neurons or neurons presynaptic to them. Nicotine challenge did not affect the mEPSC frequency of P3–P5 DNE preparations. Thus, the stimulatory effect on axon terminals at earlier stages may be blunted and/or the reduction in sEPSC frequency in P3–P5 animals reflects sites of action other than the presynaptic terminals. Although the mechanisms responsible for the qualitative change in nicotinic control of glutamatergic synaptic transmission in P3–P5 animals is unknown, we hypothesize that this is part of the compensatory response to the increased excitability in XIIMNs from nicotine exposed animals (Pilarski et al., 2011; Jaiswal et al., 2013). The P3- to P5-d delay may reflect the time needed to establish compensatory mechanisms after birth, which is accompanied by a sudden demand for tongue muscle activation to support suckling, licking, and swallowing (Thiels et al., 1990). Strategies that neural systems use to compensate for increased excitability could include reducing dendrite volume, increasing pre and/or postsynaptic inhibitory input, or reducing excitatory input. There is evidence that all three adjustments occur in XIIMNs from DNE animals. First, the dendritic tree in XIIMNs from DNE animals is larger and more complex than that of controls on P1–P2, but is smaller and less complex by P3–P4 (Powell et al., 2016), indicating changes in the timing of growth and pruning of XIIMNs due to DNE. Similarly, DNE may alter the growth and pruning of specific populations of glutamatergic neurons in this region, which could result in altered responses to acute nicotine challenge as seen here. This is supported by work showing that neuronal development is dependent on the trophic effects of ACh, both *in utero* and into adolescence, and disruption of nicotinic cholinergic signaling in this developmental window alters brain morphology and function, leading to behavioral abnormalities in both humans and animal models (Slotkin et al., 1987; Navarro et al., 1989; Slotkin, 2004). Additionally, changes in the frequency of inhibitory inputs to XIIMNs are known to occur with DNE (Wollman et al., 2018a,b), and DNE is associated with an increase in the postsynaptic response to muscimol in XIIMNs (Wollman et al., 2018a), consistent with an increase in GABA receptor expression (Jaiswal et al., 2016). Other possibilities include inhibitory effects of nAChR activation, activation of GABAB receptors, which were not blocked, or perhaps nicotine-mediated effects on other neuromodulators that inhibit glutamatergic inputs to XII motoneurons, such as serotonin (Singer et al., 1996).

### Influence of DNE on the balance of excitatory and inhibitory inputs to XIIMNs

A noteworthy finding from these experiments is that the frequency of glutamatergic sEPSCs invading XIIMNs is lower than the frequency of sIPSCs that have been observed in these cells in previous experiments. For example, glutamatergic sEPSCs in XIIMNs at baseline had an average IEI of 1500 ms, while GABAergic inputs to XIIMNs had an average IEI of 400 ms under baseline conditions (Wollman et al., 2018a). These differences carry two important implications. First, the data suggest that XIIMNs are under significant inhibition under baseline conditions. And second, it is possible that only small changes in the frequency of glutamatergic inputs have large physiologic effects. This could also indicate that the strength of excitatory inputs may be much more relevant during periods of high activity than at rest, and therefore the DNE mediated changes may become more pronounced under conditions where excitatory drive is increased. Interestingly, the reduction in glutamatergic input that is evoked by nicotine challenge in stages P3–P5 cannot be due to enhanced GABAergic or glycinergic inhibition because these were blocked in the current experiments. While we can only speculate, it is possible that nicotine evoked a distinct inhibitory effect, either by direct action or by causing the release of inhibitory neuromodulators (Singer et al., 1996; Maggi et al., 2004).

### DNE and the integrity of the neuromuscular junction

Adult mammals are said to have a high “safety factor” at the neuromuscular junction, which refers to the ability of neuromuscular transmission to remain effective even under stressful conditions, such as fatiguing contractions which can occur with elevations in airway resistance or sustained hyperpnea. It is believed that the safety factor is a presynaptic phenomenon, such that the amount of ACh released per nerve impulse exceeds the quantity needed to activate nAChRs and depolarize muscle. However, neuromuscular transmission includes postsynaptic mechanisms as well, and it is possible that DNE alters the function of nAChRs at the neuromuscular junction. Moreover, there is evidence that the safety factor in neonates is lower than in adults due to a relatively low quantal content (Kelly, 1978) and more axonal branch point failure (Fournier et al., 1991). Accordingly, we examined the integrity of the neuromuscular junction in control and DNE neonates by stimulating the phrenic nerve repetitively, with periodic superimposition of direct stimulation of muscle fibers. Comparison of the force evoked by phrenic nerve and/or direct stimulation of the muscle fibers showed that DNE had no influence on neuromuscular transmission.

An important caveat is that we used the diaphragm muscle for these experiments, rather than the tongue muscles, which are innervated by XIIMNs. Pilot studies showed that measuring force in excised tongue muscles from such small animals is difficult and we were not confident that the measured force was accurate or reproducible. The phrenic nerve-diaphragm preparation did not present these challenges, which explains why it has been an archetypal model for studying the neuromuscular junction.

### Functional significance

The consensus explanation for sudden infant death syndrome (SIDS) and apparent life-threatening events (ALTEs) is the triple risk hypothesis (Filiano and Kinney, 1994), which is based on the convergence of (1) a vulnerable neonate (typically due to risks established *in utero*); (2) a critical developmental period; and (3) an exogenous stressor. Maternal smoking is indeed the number one risk factor for SIDS and apparent life threating events in neonates, but the lack of a suitable animal model to probe the mechanisms underlying this association has been elusive. It is noteworthy that SIDS risk is highest in infants aged four to five months, while it has been suggested the first two weeks of life in the rat corresponds to 28–40 weeks of gestation in humans (Dwyer et al., 2009), so although the model mimics the triple risk model of SIDS, the developmental discrepancies between rodents and humans make an exact comparison impossible. Nevertheless, we propose that our animal model replicates, at least in part, the triple risk model. Specifically, DNE results in a decrease in nicotine-mediated glutamatergic drive to XIIMNs (vulnerability) at P3–P5, but not P1–P2 (critical developmental period), and when we imposed airway occlusion (external stressor), the DNE animals had a markedly blunted GG muscle motor response and were less likely to survive the challenge. In summary, the combination of *in vitro* and *in vivo* models used here has proven useful in the quest to understand how, and to what extent, maternal smoking/nicotine exposure impairs the function of hypoglossal motoneurons and protective respiratory reflexes.

## Synthesis

Reviewing Editor: Douglas Bayliss, University of Virginia School of Medicine Decisions are customarily a result of the Reviewing Editor and the peer reviewers coming together and discussing their recommendations until a consensus is reached. When revisions are invited, a fact-based synthesis statement explaining their decision and outlining what is needed to prepare a revision will be listed below. The following reviewer(s) agreed to reveal their identity: NONE. Note: If this manuscript was transferred from JNeurosci and a decision was made to accept the manuscript without peer review, a brief statement to this effect will instead be what is listed below.

Synthesis

This transferred manuscript was seen by one of the original reviewers, and by a new reviewer - both feel that this is an important piece of work that addresses effects of nicotine exposure during development on breathing networks. As you will see in the appended comments (below), the new review outlines a number of points that require further clarification. Of particular note, please provide some additional detail and justification for the numbers of sEPSC and mEPSC events examined for the different conditions, as there was a perception that only a very small number of events were included in the analysis. I expect that your revised manuscript will be re-reviewed by at least one of these reviewers.

Reviewer #1

GENERAL COMMENTS

Overall this is a solid manuscript that extends the work of this group and adds significantly to current literature on the effects of developmental nicotine exposure on key output neurons (hypoglossal motoneurons) of neural network that controls breathing. The main contributions of the study are nicely summarized by the previous reviewers and include:

1) activation of genioglossus muscle activity during inspiration during a 15 second airway occlusion was slowed and the incidence of nonrecoverable airway occlusion was greater in P3-5 rats that received perinatal nicotine , 2) the effect of acute nicotine exposure on synaptic activity of XII MNs was opposite in 2-5 day old rats exposed to prenatal nicotine versus controls and 3) both phrenic/diaphragm function and evoked AMPA currents were not different in treated animals at either age. Inclusion of the in vivo experiments measuring the GG EMG response to nasal occlusion greatly strengthens the study (despite, the lack the P1-2 age group for the in vivo studies). Use of the phrenic nerve - diaphragm preparation to assess whether DNE alter neuromuscular transmission is reasonable. These are challenging experiments but their overall contribution to the impact of the paper is diminished by the lack of any discussion about how these data reflect what might be happening at the XII MN to GG muscle synapse. Despite its potential significance, I have a number of questions that need to be addressed before the study can be properly evaluated. Most are issues of clarification where the either aspects of the methods/analysis or specific statements are unclear. I have also added a couple of comments related to comments of previous authors that I do not feel have been properly addressed in the revised manuscript.

MAJOR COMMENTS

Lines 44-46. The actions prenatal nicotine on brain development are not simply due to the inappropriate activation of nicotinic receptors and the subsequent modulation of synaptic transmission. Inappropriate activation of nAChRs in the fetal brain by early life exposure to nicotine serves as a switch from neuronal proliferation to differentiation and can induce apoptosis. This should be mentioned.

See Slotkin TA, Orband-Miller L, Queen KL. Development of [3 H] nicotine binding sites in brain regions of rats exposed to nicotine prenatally via maternal injections or infusions. J Pharmacol Exp Ther. 1987; 242, 232-237.

Navarro HA, Seidler FJ, Eylers JP, et al. Effects of prenatal nicotine exposure on development of central and peripheral cholinergic neurotransmitter systems. Evidence for cholinergic trophic influences in developing brain. J Pharmacol Exp Ther. 1989; 251, 894-900.

Lines 108-9. Pregnant dams were euthanized on postnatal day 5 using institutionally and federally-approved procedures.

Two things with this sentence are unclear. First, if the neonatal rat pups are at postnatal day 5, the mothers are no longer pregnant dams. Second, lines 80-81 states that rats were used between P1 and P12. If the mothers were killed at postnatal day 5, how were the pups maintained for the 7 days after the mother was euthanized. A related issue raised previously, I did not see any reference to where P12 pups were used.

Lines 174-6. “Moreover, nAChRs are located on the soma and dendrites of glutamatergic neurons, as well as on their presynaptic terminals, so exposure to nicotine could alter inputs originating from both sources.”

I am not clear what the authors are suggesting here. I understand the importance of performing analysis of sEPSCs and mEPSCs. I also appreciate that nAChRs are somal, dendritic and presynaptic. The mEPSC analysis is essential to assess pre- vs postsynaptic actions of the DNE. The statement however, implies that the analysis of sEPSCs vs mEPSCs will allow the authors to assess whether DNE alters somal inputs vs dendritic inputs; i.e. inputs originating from both sources, where the source input I assume are are dendritic or somal. I think the authors simply mean that this would help them discriminate the sites of DNE action - on pre vs postsynaptic sites. Please clarify

Lines 210-11 and 217-221. sEPSC and mEPSC actitivity were recorded under baseline conditions for 3 min and after nicotine administration for an additional 3 min. Later in the methods authors indicate that only one min of control data one minute of data during nicotine exposure were analyzed. Based on the freq of sEPSCs and mESPSCs apparent in the traces of Figs 4 and 5 respectively, this would give ~40 sEPSC events for each control animal and even fewer mEPSCs. This is a very small number of events for this type of analysis. An important addition will be to include the number of events (sEPSCs or mEPSCs) that were examined per neuron.

POSTSYNAPTIC AMPARs. I am not convinced that examining the effects of exogenous AMPA is a great way to discriminate how DNE alters postsynaptic AMPA receptor mediated synaptic transmission. Bath application of AMPA produces a slowly increasing concentration of AMPA to the bath such that the main current measured is that remaining after AMPA receptors have desensitized (i.e. the steady state AMPA current is a fraction of the peak). Also, this process activates extrasynaptic AMPARs and synaptic...Whereas it is largely the synaptic AMPA receptors that will influence synaptic transmission. The sEPSC and especially the mEPSC data that show no change in amplitude are better measurements. The only issue here is related to that raised above, specifically the low number of PSCs analyzed.

Similarly:

Lines 457-61. “We also analyzed the amplitude of sEPSCs and mEPSCs, and because differences in the amplitude of spontaneous events in control and DNE cells could be due to either presynaptic or postsynaptic mechanisms (Vautrin and Barker, 2003), we assessed postsynaptic receptor function directly with bath application of AMPA in synaptically isolated neurons.”

The analysis of mEPSC amplitude is a better way to assess postsynaptic AMPA receptor properties than responses to exogenously applied AMPA. The former examines responses to exogenous glutamate most likely at synaptic AMPA receptors. Bath application of AMPA will activate extrasynaptic and synaptic receptors and the two populations may respond differently to DNE and acute nicotine. This needs to be discussed.

Line 427, The main finding is that in DNE pups aged 3-5 days, nicotine challenge caused a marked slowing of excitatory synaptic input to XIIMNs.“

Authors need to clarify this statement. DNE caused a marked delay (not a slowing) in the GG EMG response to airway occlusion. A marked slowing of excitatory synaptic input could be incorrectly interpreted as indicating that the kinetics of the glutamatergic signaling has slowed.

Lines 434-436. ”...underlie the observation that DNE blunts the genioglossus motor response to nasal occlusion and makes these animals more susceptible to failed auto-resuscitation.“

This statement requires a reference supporting that DNE makes the animals more susceptible to suffer auto-resuscitation failure. Authors did not perform a rigorous analysis of auto-resuscitation in this study - only that more of the DNE animals died. GG EMG activity is not equivalent to autoresuscitation.

Data in Figure 4. It is not clear how nicotine challenge in the DNE mice could lead to an increase in the IEI (i.e., a decrease in the frequency) of sEPSCs. Authors suggest this is due to enhanced GABAergic transmission but GABA signaling is blocked in these experiments.

Nicotine, through nAChRs must be having a direct inhibitory action on the frequency of sEPSCs. Authors also suggest “Instead, these observations (the increased IEI) are consistent with long-lasting desensitization of nAChRs on glutamatergic neurons that synapse onto XIIMNs, as is known to occur in other brain regions following chronic nicotine exposure (Gentry and Lukas, 2002).”

I do not understand this proposed mechanism in the context of the data presented. If the decrease in sEPSC was simply due to desensitized nAChRs on premotoneurons, this would require that activation of non-desensitized nAChRs on premotoneurons from non-nicotine exposed animals would increase sEPSCs. Desensitization of nAChRs could result in loss of a nicotine evoked increase in sEPSCs. However, this is not what was observed. Acute nicotine treatment has no effect on sEPSCs in control slices. Thus, there is no excitatory nAChR mechanism available to desensitize. Activation of nAChRs in the DNE group reduced the frequency of sEPSCs. With inhibitory transmission blocked, the mechanism underlying this action of nicotine is not clear. Some hints might come from work in nucleus accumbens synaptosomes, but this is also highly speculative. See review by Marchi et al. 2015. Nicotinic modulation of glutamate receptor function at nerve terminal level: a fine-tuning of synaptic signals, Frontiers in Pharmacol; doi: 10.3389/fphar.2015.00089.

Fig 3. It is not at clear what is going on in panel A. Where does the occlusion start or stop? The caption indicates that this shows breathing difficulties after occlusion but it is not clear where the occlusion started or stopped.

The PSC recordings in Figs 4 and 5 are extremely brief and on their own do not allow one to properly evaluate the effects of nicotine on PSC properties (frequency and amplitude). These shorter segments should be included but as excerpts from longer duration recordings.

Comments from previous reviewers

Author response to previous reviewer comment: “We also have no reason to believe that the neuromuscular junctions would vary significantly across muscles, though we have acknowledged that they may.”

While the neuromuscular junctions may not vary significantly across muscles, the impact of nicotine exposure on MN development and the pattern of muscle innervation may indeed differ between muscles... This potential should at least be mentioned in the discussion. Authors have acknowledged in their response that the neuromuscular junctions might vary across muscles, they have not done so in the manuscript.

Author response: “Body temperature was kept constant through these experiments using a temperature probe and heat lamp. Influences of maternal separation should affect control and nicotine-exposed pups the same, though we now acknowledge that there could be a difference”.

This caveat regarding potential thermal stress and maternal separation needs to be included because the effects of stress may be different in control vs nicotine exposed animals. Authors indicate in their rebuttal that they “now acknowledge that there could be a difference (between control and DNE mice)”. However, I am unable to find any mention of maternal separation and that this may differ for the control and DNE treated animals.

Comment of previous reviewer: “More thorough discussion of the basis for differences between 1-2 versus 3-5 days is warranted. ”

There is no discussion of the potential mechanism by which this developmental difference would arise. Given the early presentation of nicotine during gestation, what about DNE might account for a sudden change between P1-2 and P3-5?

Comment of previous reviewer. The authors explain their findings of decreased frequency of sEPSCs after a nicotine challenge in DNE pups with increased GABAergic inhibitory tone.

Authors have not addressed this point. While overall DNE may result in a shift toward increased GABAergic tone, this shift can not explain the effects of acute nicotine exposure presented in this manuscript because GABAergic signaling has been blocked. Thus, at this point in the manuscript authors simply need to clarify that they are referring to global changes, not the results obtained in the present study where GABAergic influences were blocked; i.e., I did not get the impression that authors were attributing the changes they report in this paper to increased inhibition. Still, this should be clarified. Authors should at least discuss this issue and mention that data re the effects of nicotine challenge on sEPSCs was the same as in Wollman et al. 20181)

Comment of previous reviewer. Authors state that a P3 rat pup is comparable to a premature fetus at 23 weeks of gestation. They should include a brief discussion of how the age of the rats studied compares with human development and SIDS risk. SIDS risk is highest postnatally (~4 months of age). The developmental changes in the impact of DNE on motoneuron excitability are very interesting but it is hard to envision that if P3 in the rat corresponds to the 23rd week of human gestation that p5 corresponds to a 4 month old infant when the risk for SIDS is highest. Authors should discuss the challenge of comparing brain developmental stages between species.

MINOR COMMENTS

Lines 83-84. The ambiguity of this sentence was pointed out previously and it has not been corrected.

“An equal number of control and nicotine exposed animals were used from each litter.”

This is not possible since in nicotine exposed litters, all littermates were exposed.

Lines 151-153. The EMG activity was normalized in each animal by first detecting the largest burst recorded during the experimental procedure and assigning a value of 1.0 to that burst.

If GG of the DNE animals is always lower than in controls, this analysis procedure biases the data towards not detecting a difference between control and DNE groups. This should be mentioned.

Line 193. MW should be Mohms.

Line 207. Please refer the reader to the table for drug concentrations of AP5, strychnine and bicuculline.

Line 236.“Nicotine was used at the highest dose that produced presynaptic effects without producing a significant inward current, which we found to be 0.5 μM.”

Please indicate how authors define that this concentration produced a presynaptic effect.

Line 239. “AMPA was used at a concentration that produced...”

Please provide the concentration here.

Line 302 - please indicate here the average number of events (sEPSCs or mEPSCs) analyzed per neuron.

Line 323. Instead of Remmers 2001, this should cite the original paper of Remmers from 1978. This same paper should be cited in the introduction.

Line 343. “Interestingly, of the 12 neonates that entered this state of unstable breathing and airway obstruction...”

The reference to airway obstruction here should be removed. In the previous sentence authors state that this instability likely reflects obstructive apnea and explain why. This is fine. It is not ok, however, to stipulate in the next sentence that these events ARE obstructions.

Line 480-81. Similar to the comment above, this sentence “...indicating that the slowing of sEPSCs...” needs to be clarified. The interevent interval has increased so the freq has decreased but this is not the same as saying that the sEPSCs have slowed down.

Lines 502-3. “...high ”safety factor“ at the neuromuscular junction, which refers to the ability of neuromuscular transmission to remain effective even under very stressful conditions.”

Please define what is meant by “very stressful” in the context of motoneuron and muscle activity levels.

Line 506. Change to “depolarize the muscle cell to threshold”.

Some discussion is required regarding the possibility that DNE might differentially affect XII vs phrenic MNs and their respective neuromuscular junctions.

Table 3. The RN values are higher than expected for MNs using standard gluconate based solutions but these higher values are expected based on the use of a Cs based intracellular solution. This should be mentioned somewhere in the manuscript.

Also, there is no point in reporting RN valuies to the first decimal point when the SE is in the range of 10s of MOhms.

The same applies to the error values in the measurement of Vm (meaningless to report to 0.01 mV).

Figure 1A. The mouse in the diagram and the EMG wires should be improved to a professional quality. Also, there is no EMG lead connected to the diaphragm.

Fig 1B. The EMG traces would be complemented with integrated recordings as this would better show the gradually incrementing amplitude of the bursts during the nasal occlusions. In addition, given the marked differences in the response of P1-2 and P3-5 neurons DNE motoneurons to acute nicotine challenge and the suggested link between this altered excitability and the reduced EMG responses of the DNE mice to airway occlusion (and their greater incidence of death following nasal occlusion), it would have been a great addition to the study for Figure 1 and 2 to have included this same age group comparison (rather than just the analysis of P3-5 animals in vivo). The reason for not doing this should be mentioned.

The trace in figure 6A is very low resolution, The PSCs can hardly be discriminated.

Also the bar indicating AMPA application appears right-shifted. As illustrated, the AMPA currents starts to recover toward baseline while the AMPA is still on. I understand the delay between AMPA onset and the start of the inward current (circulation time and diffusion will delay the response). However, the same factors, removal of AMPA from the bath, will take time after AMPA is removed so I would expect the AMPA current to start to recover AFTER the AMPA application has stopped.

Fig 7A, Indicate on the figure that this is nerve stim.

Reviewer #2

The authors have generally responded satisfactorily to my previous review. There were one concern remaining:

• Previous comment: “An equal number of control and nicotine exposed animals were used from each litter.” This cannot be true since all animals within a given litter received either nicotine or vehicle. There is no way to split the litter in the way that this sentence states.
